# The gut-eye axis in inflammatory bowel disease: shared immune mechanisms and targeted therapies

**DOI:** 10.3389/fimmu.2026.1997593

**Published:** 2026-10-26

**Authors:** Xuewei Fu, Shisi Hu

**Affiliations:** 1 Department of Emergency, Hainan General Hospital, Hainan Affiliated Hospital of Hainan Medical University, Haikou, China; 2 Hainan Eye Hospital and Key Laboratory of Ophthalmology, Zhongshan Ophthalmic Center, Sun Yat-sen University, Haikou, China

**Keywords:** inflammatory bowel disease, extraintestinal manifestation, gut-eye axis, episcleritis, uveitis, ocular disease

## Abstract

Inflammatory bowel disease (IBD) is recognized not only as a disease confined to the intestinal tract, but also as a systemic immune-mediated disorder. Among its diverse extraintestinal manifestations, ocular complications, which range from self-limiting episcleritis to severe and sight-threatening uveitis and scleritis, represent a critical clinical challenge that profoundly impacts patient quality of life. This review synthesizes the epidemiological landscape and clinical spectrum of ocular manifestations in IBD, framing their pathogenesis through the emerging concept of the gut-eye axis. We critically examine the shared mechanistic pathways driving this bidirectional relationship, including the systemic dissemination of circulating inflammatory cytokines, aberrant lymphocyte homing, cross-reactive autoantibodies, shared genetic susceptibilities, and microbiota dysbiosis, which collectively compromise the blood-ocular barrier. Furthermore, we outline targeted therapeutic strategies, highlighting that while systemic anti-TNF agents remain the cornerstone for severe and refractory ocular inflammation, gut-selective biologics like vedolizumab necessitate careful application due to the risk of extraintestinal exacerbation. Finally, we underscore the indispensable role of multidisciplinary management and highlight future research trajectories.

## 1 Introduction

Inflammatory bowel disease (IBD), principally comprising Crohn’s disease (CD) and ulcerative colitis (UC), represents a spectrum of chronic, immune-mediated inflammatory disorders of the gastrointestinal tract. Its pathogenesis is highly complex, driven by a convergence of genetic susceptibility, environmental triggers, microbiota dysbiosis, epithelial barrier defects, and fundamentally dysregulated innate and adaptive immune responses (1, 2). Increasingly, IBD is recognized not only as a disease confined to the intestinal tract, but also as a systemic immune-mediated disorder. Up to 50% of patients develop at least one extraintestinal manifestation (EIM) during their disease course, with EIMs even preceding gastrointestinal symptoms in a significant proportion of patients (3). These manifestations most frequently target the joints, skin, hepatobiliary-pancreatic system, and eyes. The clinical trajectory of EIMs is highly variable; they may parallel intestinal inflammatory activity (e.g., peripheral arthritis, erythema nodosum, episcleritis) or progress entirely independent of bowel symptoms (e.g., anterior uveitis, ankylosing spondylitis, primary sclerosing cholangitis). Consequently, EIMs profoundly impact patient quality of life and often dictate the overall therapeutic strategy (4).

Among these systemic complications, ocular manifestations represent a relatively common category of EIMs, often reported after joint and cutaneous involvement, and affect approximately 1% to 7% of patients with IBD in most cohorts. The clinical spectrum is broad, ranging from benign, self-limiting episcleritis to severe, sight-threatening conditions such as uveitis and scleritis. Notably, ocular EIMs exhibit a higher predilection for CD than UC. They are frequently associated with female sex, the presence of concurrent EIMs, and a generally more aggressive IBD phenotype that necessitates increased hospitalizations and reliance on systemic corticosteroids or biologic therapies (3, 5–8). Indeed, the pathophysiological link between intestinal and ocular inflammation is increasingly conceptualized through the “gut–eye axis”. The gastrointestinal tract harbors the body’s largest immune compartment, with 70–80% of systemic immune cells residing within the gut-associated lymphoid tissue (GALT), Peyer’s patches, and the lamina propria. Under physiological conditions, commensal microbes continuously educate this mucosal immune network, maintaining homeostasis through the delicate balancing of effector (e.g., Th17) and regulatory (Treg) T cells (9). However, microbiota dysbiosis and loss of epithelial barrier integrity, both hallmarks of IBD, may facilitate the systemic dissemination of microbial products and inflammatory mediators (10). This breach can initiate systemic inflammatory cascades and aberrant immune cell trafficking to distant sites, thereby promoting ocular inflammation through blood-ocular barrier disruption, altered immune cell homing, and impairment of local ocular immune tolerance.

The present review aims to provide a comprehensive and clinically oriented synthesis of the epidemiology, clinical spectrum, and therapeutic management of ocular manifestations in IBD, alongside mechanistic insights into the gut-eye axis, highlighting implications for multidisciplinary care and future research directions.

## 2 Search strategy

This article is a narrative review and was not designed or conducted as a systematic review. A literature search was primarily conducted in the PubMed database for articles published from its inception to September 30, 2026. We used the Medical Subject Heading (MeSH) terms and keywords in all possible combinations using Boolean operators with the following search strategies: “inflammatory bowel disease”, “Crohn’s disease”, “ulcerative colitis”, “eye manifestations”, “eye”, “ocular”, “ophthalmic”, “uveitis”, “episcleritis”, “scleritis”, “iritis”, “iridocyclitis”, “dry eye”, “keratopathy”. To ensure literature saturation, the reference lists of the retrieved full-text articles and previous systematic reviews were also manually screened. The search was restricted to articles published in the English language.

## 3 Epidemiology and clinical spectrum of ocular manifestations in IBD

Due to the relatively low incidence of ocular EIMs in IBD, small-cohort studies are inherently prone to selection bias and wide margins of error. Therefore, defining the true epidemiological landscape requires large-scale data from population-based registries, administrative claims databases, and multicenter cohorts (**Table 1**), and comprehensive meta-analyses. A recent major meta-analysis encompassing over 350,000 patients highlighted the high systemic burden of the disease, demonstrating that at least one major EIM (joint, ocular, or skin) occurs in 24% of the overall IBD population, with a higher frequency in CD than in UC (11). Within this broader context, a synthesis of large-cohort studies reveals that the overall prevalence of specifically ocular EIMs typically ranges from 1% to 7%, heavily depending on the study design and the specific patient population. Recent large-scale longitudinal tracking has established an incidence rate of 2.9 per 1,000 person-years (8). Across nearly all major databases, patients with IBD exhibit a significantly elevated risk of developing ophthalmic conditions compared with the general population.

**Table 1:** Large-cohort studies reporting ocular manifestations in IBD.

Author, year, country	Study design	Sample size	Control size	Case definition	Main findings
Bernstein et al., 2001, Canada (12)	Population-based retrospective cohort study	IBD: 4,454	44,540	Diagnostic codes (ICD-9-CM)	1. Iritis/uveitis was the most common EIM (prevalence: CD: 1.5%, UC: 2.0%). 2. Patients with IBD had a significantly higher risk of ocular EIMs than the general population (female: OR, 6.3; 95% CI, 3.9-10.3; male: OR, 3.2; 95% CI, 1.6-6.4).
Jose et al., 2009, United States (13)	Multicenter registry cohort study	IBD: 1,649	N/A	Clinical diagnosis with specialist confirmation	1. Ophthalmologic conditions comprised 7.0% of all initial EIMs. 2. The 10-year cumulative incidence of iritis following IBD diagnosis was 1.8%.
Vavricka et al., 2015, Switzerland (14)	Multicenter cohort study	IBD: 1,249	N/A	Clinical diagnosis with ophthalmologist confirmation	1. Uveitis accounted for 13.7% of all EIMs in the cohort. 2. Over half of the uveitis cases (52.2%) were diagnosed prior to the underlying IBD diagnosis.
Card et al., 2016, United Kingdom (15)	Cross-sectional study	IBD: 56,097	280,382	Diagnostic codes (Read codes)	1. Uveitis was present in 3.4% of the overall IBD patient cohort. 2. IBD patients had a significantly higher risk of uveitis compared to the general population (OR, 2.81; 95% CI 2.65-2.98).
Karmiris et al., 2016, Greece (4)	Multicenter retrospective cohort study	IBD: 1,860	N/A	Clinical diagnosis with ophthalmologist confirmation	Ocular EIMs were observed in 3.0% of IBD patients.
Yang et al., 2018, Korea (16)	Population-based cross-sectional study	IBD: 43,281	1,127,261	Diagnostic codes (ICD-10)	Iridocyclitis was the most common ocular EIM (prevalence: CD: 0.8%, UC: 1.2%).
Biedermann et al., 2019, Switzerland (17)	Prospective multicenter observational cohort study	IBD: 3,266	N/A	Clinical diagnosis recorded via standardized physician questionnaires	The presence of uveitis was strongly associated with a severe disease course.
Vadstrup et al., 2020, Denmark (18)	Nationwide registry-based cohort study	IBD: 32,446	32,446	Diagnostic codes (ICD-10)	1. Iridocyclitis prevalence was 1.3% in CD and 0.9% in UC. 2. IBD patients exhibited a significantly higher risk of iridocyclitis compared to the matched control population (CD: OR, 2.87; 95% CI, 2.05-4.02; UC: OR, 2.32; 95% CI, 1.79-3.00). 3. Ocular EIMs frequently preceded the actual IBD diagnosis.
Algaba et al., 2020, Spain (5)	Multicenter cross-sectional study	IBD: 31,077	N/A	Clinical diagnosis with ophthalmologist confirmation	1. Ocular EIMs were present in 2.1% of IBD patients. 2. The risk of developing ocular EIMs was significantly higher in females and patients with CD.
Lin et al., 2021, Taiwan, China (19)	Population-based retrospective cohort study	IBD: 4,508	18,032	Diagnostic codes (ICD-9-CM)	IBD patients had a significantly higher risk of developing ischemic optic neuropathy compared to those without IBD (HR, 4.135; 95% CI, 1.312-11.246).
Singeap et al., 2021, Romania (20)	Cross-sectional registry study	IBD: 2,626	N/A	Clinical diagnosis recorded	The prevalence of ocular EIMs was 1.03%.
Ko et al., 2023, Taiwan, China (21)	Nationwide cohort study	IBD: 54,293	54,293	Diagnostic codes (ICD-9-CM) and ophthalmologist confirmation	1. IBD was an independent risk factor for dry eye disease. 2. IBD patients had a significantly increased risk of corneal surface damage (HR, 1.13; 95% CI, 1.03-1.24).
Arzbecker et al., 2024, United States (22)	Single-center retrospective study	IBD: 1,320	N/A	Diagnostic codes (ICD-10) and ophthalmologist confirmation	Uveitis was present in 3.2% of the overall IBD patient cohort.
Makdoumi et al., 2024, Sweden (23)	Population-based case-control study	IBD: 19,663	39,326	Diagnostic codes (ICD-10)	1. Patients with UC had a significantly increased risk of developing eye diseases compared to controls (HR, 1.25; 95% CI, 1.20-1.32). 2. The highest increased risk was seen in anterior segment inflammatory conditions such as episcleritis, keratitis, and anterior uveitis.
Maniscalco et al., 2024, Italy (24)	Multicenter retrospective cohort study	IBD: 4,229	N/A	Clinical diagnosis with specialist confirmation	The overall prevalence of chronic uveitis in pediatric IBD was 141.8 per 100,000 patients.
Ben-Tov et al., 2025, Israel (25)	Population-based cross-sectional study	IBD: 2,567	N/A	Diagnostic codes (ICD-9)	Ocular manifestations were present in 3.0% of the pediatric IBD cohort.
Yanai et al., 2026, Israel (8)	Population-based retrospective cohort study	IBD: 15,035	N/A	Diagnostic codes (ICD-9)	1. The overall incidence rate of ocular manifestations was 2.9 per 1,000 person-years. 2. Uveitis was the most common ocular manifestations diagnosis. 3. Increased risk of ocular manifestations was associated with markers of severe disease.

Regarding the clinical spectrum, anterior segment inflammation remains the hallmark of IBD-related ocular involvement. Iritis, uveitis (particularly anterior uveitis/iridocyclitis), and episcleritis are consistently identified as the most frequent diagnoses across global cohorts (12, 16, 23). Beyond these classical inflammatory lesions, recent nationwide data have broadened the known spectrum of IBD-associated ocular morbidity to include an increased risk of dry eye disease, corneal surface damage, and ischemic optic neuropathy (19, 21). Furthermore, smaller cohorts and case reports have documented rare but severe posterior segment and neuro-ophthalmic complications, such as central retinal vein occlusion, retinal vasculitis, branch retinal artery occlusion, and optic neuritis (26–29). Overall, advanced ophthalmic imaging shows that IBD patients often have subclinical retinal, choroidal, corneal, and ocular-surface abnormalities even without overt ocular inflammation or visual symptoms, but the direction and disease-activity relevance of these changes are inconsistent across studies (30–35).

The development of ocular EIMs is closely tied to specific demographic and clinical factors. The risk is generally higher in patients with CD compared with those with UC and frequently demonstrates a female predominance (5, 17, 36). Crucially, the emergence of ocular complications serves as a clinical indicator of systemic inflammatory burden. Ocular involvement is strongly associated with a more severe and refractory IBD phenotype, often requiring aggressive medical or surgical interventions, including systemic corticosteroids, biologics, or intestinal resection (7, 8, 17).

A critical chronological characteristic of IBD-related ocular EIMs is their potential to precede gastrointestinal symptoms. Strikingly, multiple cohorts have demonstrated that ocular inflammation can frequently occur prior to the actual diagnosis of IBD. A multicenter cohort of 1,249 patients demonstrated that in 52.2% of uveitis cases, ocular inflammation preceded the diagnosis of the underlying IBD (14). Similarly, individuals presenting with idiopathic uveitis have a significantly higher cumulative incidence of subsequent IBD, with one study noting a 1.49-fold increased risk of incident CD (37). However, these findings describe the timing of EIMs relative to the clinical diagnosis of IBD and do not establish that ocular inflammation precedes the biological onset of intestinal disease, which may be clinically silent. Thus, ocular inflammation may represent a clinical clue prompting evaluation for possible underlying IBD, particularly in patients with suggestive gastrointestinal or systemic features.

These epidemiological trends and risks extend to pediatric populations, where vigilance for sight-threatening conditions is equally critical. In a large multicenter cohort of pediatric IBD patients, ophthalmologic conditions accounted for 7% of all initial EIMs. Iritis emerged as the most common ocular manifestation, with its cumulative incidence steadily increasing post-IBD diagnosis (0.5% at 1 year, 1% at 5 years, and 1.8% at 10 years) (13). Other large pediatric registries, including the Italian SIGENP cohort and population-based cross-sectional studies, report an overall ocular involvement prevalence of approximately 3%, with episcleritis, scleritis, and chronic uveitis being the primary presentations (24, 25). These longitudinal findings indicate a continuous risk of ocular complications throughout the disease course, emphasizing the necessity of incorporating systematic ophthalmologic evaluations into the long-term clinical monitoring of children with IBD.

While the epidemiology and clinical spectrum of ocular manifestations in IBD are increasingly well described, these estimates must be interpreted with caution. Most IBD cohorts were not primarily designed to investigate ocular EIMs and rarely incorporated standardized, prospective ophthalmologic screening. Ascertainment has typically depended on patient-reported symptoms, subsequent referral to ophthalmology, or diagnostic coding in administrative databases. Consequently, mild, episodic, or self-limiting inflammation (e.g., episcleritis) may be underreported, while subclinical or minimally symptomatic uveitis could remain undetected without routine slit-lamp examinations. Conversely, referral-based studies may overestimate the burden of severe ocular inflammation. Ultimately, standardized definitions, ophthalmologist-confirmed diagnoses, and longitudinal IBD–ophthalmology-linked cohorts are still required to accurately define the true burden, temporal patterns, and prognostic implications of ocular EIMs.

## 4 Pathogenesis of ocular involvement in IBD

Although precise mechanisms remain unclear, IBD-associated ocular pathogenesis is multifactorial, arising from an interplay of systemic and local mechanisms via the gut-eye axis. The translation of intestinal inflammation to ocular tissue damage is thought to be mediated by the systemic dissemination of circulating inflammatory mediators that compromise the blood-ocular barrier, coupled with suspected adaptive immune responses involving autoantibodies and shared cross-reactive antigens. This process may be further amplified by the aberrant homing of intestine-primed lymphocytes, and appears to be underpinned by genetic susceptibilities.

### 4.1 Immune dysregulation and circulating inflammatory mediators

Disruption of intestinal epithelial tight junctions and the mucus barrier in IBD permits luminal antigens to breach the intestinal epithelium, activating dendritic cells and macrophages via pattern-recognition receptors, such as Toll-like receptors. This initial innate immune activation triggers a robust release of pro-inflammatory cytokines, most notably tumor necrosis factor-alpha (TNF-α), interleukin (IL)-1β, IL-6, and IL-23. Rather than remaining confined to the gut mucosa, these locally produced mediators enter the systemic circulation, establishing a systemic pro-inflammatory state (38–40). Consequently, this circulating inflammatory milieu can contribute to the pathogenesis of diverse ocular diseases through shared immune-mediated and oxidative stress pathways (41, 42). Supporting this premise, epidemiological evidence from a large UK Biobank cohort demonstrated that systemic immune-inflammation indices are significantly and dose-dependently associated with an increased risk of various incident ocular disorders over a 13-year follow-up, underscoring the profound impact of systemic inflammation on ocular health (43).

The translation of systemic inflammation into localized ocular pathology likely hinges on the disruption of the blood-ocular barrier in IBD. In the inflamed gastrointestinal tract, cytokines such as IFN-γ drive pathogenesis by breaking down the vascular barrier through the disruption of the adherens junction protein VE-cadherin; notably, endothelial-specific knockout of the IFN-γ receptor ameliorates experimentally induced colitis (44). It is highly plausible that similar cytokine-driven mechanisms compromise the vascular barriers of the eye. Indeed, evidence indicates that systemic cytokines, including TNF-α, IL-1β, and IL-17A, directly impair the integrity of the blood-ocular barrier (45–47). Conversely, certain pathways may exert a protective effect. For example, IL-36R signaling enhances adherens and tight junctions, induces vasculoprotective processes, and drives vessel remodeling and stabilization in the retinal vasculature (48).

Convergent mechanisms involving circulating systemic inflammation, oxidative stress, and hypoxia can further disrupt tight junction proteins at the blood-retinal and blood-aqueous barriers. The weakening of these tight junctions between retinal pigment epithelial cells or uveal vascular endothelial cells allows for the anomalous influx of fluid, serum proteins, lipids, and circulating inflammatory mediators into the neural and uveal extracellular spaces. This structural compromise not only produces clinical manifestations like macular edema and vision loss but also promotes aberrant angiogenesis and self-perpetuating cycles of neuroinflammation (49). Ultimately, this cytokine-mediated barrier breakdown leads to the impairment of local ocular immune tolerance, transforming the classically immune-privileged eye into a vulnerable target for systemic autoimmune responses (50). However, it must be emphasized that the current literature directly investigating the specific pathogenic mechanisms of IBD-related ophthalmic manifestations remains exceedingly sparse. A critical review of the existing body of evidence reveals that the vast majority of publications are predominantly correlational or epidemiological in nature. Consequently, our current mechanistic understanding is largely extrapolated from analogous systemic autoimmune conditions rather than primary IBD research. To bridge this gap, the focus should shift from association studies to targeted translational research. Specifically, analyzing aqueous humor cytokines in patients with IBD and employing IBD-specific animal models with detailed ocular phenotyping are essential to clarify how the gut-eye axis functions.

### 4.2 Shared antigens, autoantibodies, and aberrant lymphocyte homing

Beyond the non-specific effects of circulating inflammatory mediators, the pathogenesis of ocular involvement in IBD has been hypothesized to involve adaptive immune cross-reactivity, potentially mediated by shared antigenic epitopes between the intestinal mucosa and ocular tissues. A classic paradigm of this concept involves a specific peptide epitope that is characteristically identified by the monoclonal antibody 7E12H12 and is concurrently expressed in the colonic epithelium, non-pigmented ciliary epithelial cells, and chondrocytes. The restricted anatomical distribution of this cross-reactive antigen precisely mirrors the triad of intestinal, ocular, and joint inflammation frequently observed in patients with UC (51, 52). This shared antigenicity provides a compelling biological rationale for the hypothesis that autoantibodies, initially generated against damaged colonic mucosa, can erroneously target specific ocular structures.

The generation of circulating autoantibodies against these shared organ-specific antigens remains a widely proposed mechanism for EIMs in IBD (53). Clinical data robustly support this concept; for instance, anti-neutrophil cytoplasmic antibody (ANCA) seropositivity is significantly associated with the overall presence of EIMs, particularly in CD (36). Furthermore, in a Hungarian cohort of 689 IBD patients, positivity for pancreatic autoantibodies correlated with penetrating disease behavior, perianal disease, and specific EIMs including arthritis, ocular, and cutaneous involvement (54). Furthermore, the high concurrent incidence of uveitis and CD observed in patients with psoriatic arthritis strongly reinforces the concept of a shared “gut-eye-joint” axis (55). While these clinical correlations firmly link autoantibody production to IBD-associated EIMs, the underlying evidence remains predominantly associative. The direct mechanistic translation requires further *in vivo* validation.

Parallel to humoral immunity, the paradigm of aberrant lymphocyte homing along the gut–target organ axis has offered pivotal insights into the mechanisms underlying EIMs. This concept posits that intestine-primed immune cells can translocate to distant sites and drive local pathological progression (56–58). The α4β7 integrin is expressed on subsets of activated lymphocytes, particularly gut-imprinted T and B cells, whereas its principal ligand, mucosal addressin cell adhesion molecule-1 (MAdCAM-1), is predominantly expressed on endothelial cells in gut-associated lymphoid tissues and the intestinal mucosa. The α4β7–MAdCAM-1 interaction promotes lymphocyte adhesion to the endothelium and transmigration into intestinal tissues (59, 60). Emerging evidence suggests that this pathway may also contribute to gut–retina immune crosstalk under pathological conditions. Studies demonstrate that elevated intraocular pressure triggers the generation of gut-homing β7^+^CD4^+^ T cells, which undergo transcriptional reprogramming in the gut before infiltrating the retina via MAdCAM-1, ultimately driving progressive retinal ganglion cell degeneration (61). Similarly, reinforcing the bidirectional nature of this gut-retina axis, a recent study revealed that MAdCAM-1 is aberrantly upregulated in retinal microvessels during experimentally induced colitis. This ectopic expression directly recruits gut-homing CD4^+^ T cells to the retina, where they co-localize with bipolar and retinal ganglion cells to drive neurodegeneration (62). Thus, this aberrant homing process acts as a central pathogenic pathway, in which extraintestinal sites like the eye aberrantly express adhesion molecules that inappropriately recruit gut-homing memory lymphocytes generated during bowel inflammation.

### 4.3 Genetic predisposition and intestinal microbiota

Genetic susceptibility to ocular EIMs in IBD involves both HLA and non-HLA pathways. Clinical evidence has firmly linked specific alleles, including HLA-B27, HLA-B58, and HLA-DRB1*0103, to ocular inflammation. Furthermore, a large multicohort analysis reinforced the critical role of this genetic locus by identifying a genome-wide significant association within the major histocompatibility complex (36, 63). HLA-B27 is the strongest genetic risk factor for acute anterior uveitis. Although it is not a primary genetic driver of IBD itself, HLA-B27 represents the strongest genetic determinant of axial spondyloarthritis and ankylosing spondylitis occurring as EIMs of IBD (64, 65). Recent large-scale genome-wide studies have further solidified this shared genetic architecture, demonstrating significant genetic correlations and supporting a causal effect of IBD on anterior uveitis risk (66). Beyond the HLA region, non-HLA genetic factors also contribute significantly. The *NOD2/CARD15* risk variant, the first identified genetic driver in CD, is concurrently associated with uveitis, suggesting a shared pathogenic mechanism (67). Additionally, a polymorphism in the autophagy-related gene *ATG16L1* confers a dual risk for both CD and uveitis, implicating defective macrophage-mediated autophagy as a molecular bridge between intestinal and ocular inflammation (68, 69). Together, this shared genetic architecture suggests that intestinal and ocular inflammation may involve overlapping immunological pathways rather than being entirely isolated events.

Beyond genetic susceptibility, gut microbiota dysbiosis promotes various ocular conditions through systemic immune dysregulation, altered microbial metabolite signaling, and compromised barrier function (70–72). Disruption of the intestinal barrier facilitates the systemic translocation of microbial products and gut-primed immune cells, which subsequently migrate to immunologically privileged sites such as the eye, triggering uveitis (73, 74). *In vivo* models demonstrate that maintaining a germ-free environment or administering broad-spectrum oral antibiotics significantly delays the onset and attenuates the severity of experimental autoimmune uveitis (75). While direct cause-effect relationships remain complex, restoring microbial balance and its associated metabolites offers significant therapeutic potential. For example, microbiome-derived short-chain fatty acids (SCFA) have been shown to ameliorate uveitis severity by enhancing the differentiation and suppressive activity of regulatory T cells, thereby dampening the hyperactive ocular immune response (76). Gnotobiotic studies support a causal role of gut dysbiosis in autoimmune uveitis. In spontaneous experimental autoimmune uveitis mice, more severe disease was associated with *Akkermansia* expansion, depletion of SCFA-producing *Firmicutes*, reduced SCFA levels, and enhanced intestinal Th1 responses; *Akkermansia* enrichment further aggravated uveitis. An increased *Verrucomicrobia*/*Firmicutes* ratio has also been reported in uveitis, CD, and multiple sclerosis (77). Furthermore, altered microbiomes and associated metabolic pathways are implicated in the pathogenesis of major retinal vascular diseases, though direct causality requires further investigation (78). Interestingly, this gut-eye communication appears to be bidirectional. Recent studies reveal that retinal injuries, such as laser burns or detachments, rapidly alter gut microbial profiles (e.g., decreased *Faecalibacterium* and increased *Akkermansia*), increase gut permeability, and activate circulating innate immune cells. Antibiotic interventions in these models attenuated gut permeability and reduced the severity of retinal pathologies, confirming the existence of a dynamic, responsive retina-gut axis during wound healing (79). Beyond the lower gastrointestinal tract, microbial infections in other remote niches can also exert indirect ocular effects. For instance, gastric *Helicobacter pylori* infection has been associated with disrupted tear secretion and secondary alterations to the ocular surface microbiota, potentially contributing to dry eye pathogenesis via systemic immune responses and lacrimal gland inflammation (80). Furthermore, within the low-biomass microenvironment of the ocular surface, specific shifts in ecological interactions rather than major compositional changes correlate significantly with clinical improvements in meibomian gland dysfunction and dry eye symptoms (81).

The intricate interplay between genetic susceptibility and the gut microbiota represents a crucial pathogenic mechanism. A striking example of gene-microbiome interplay is the Crumbs homolog 1 (*CRB1*) gene, associated with retinal degenerations like Leber congenital amaurosis. *CRB1* mutations dampen the gene’s expression in both the retinal pigment epithelium and colonic enterocytes. The consequent impairment of both the outer blood-retinal barrier and the colonic intestinal epithelial barrier leads to the translocation of intestinal bacteria to the retina, driving secondary retinal degeneration. Crucially, systemic bacterial depletion or colonic reintroduction of normal *CRB1* expression can rescue this mutation-associated retinal degeneration, highlighting a profound link between genetic barrier defects and microbiota-driven ocular pathology (82). Furthermore, this interplay is deeply embedded in immune recognition, perhaps best illustrated by the phenomenon of molecular mimicry. Microbial components possessing cross-reactive antigens can induce autoimmune uveitis by sharing epitopes with retinal proteins like S-antigen (83). HLA-B27-associated acute anterior uveitis involves public T cell receptors that recognize shared binding motifs present in both self-antigens and microbial antigens, with structural analysis confirming TCR cross-reactivity rooted in molecular mimicry. Self-peptides derived from the iris and ciliary body are among the targets of these cross-reactive TCRs, directly linking shared antigen recognition to anterior ocular inflammation (84).

In summary, the pathogenesis of ocular EIMs in IBD is driven by an intricate triad of genetic susceptibility, intestinal dysbiosis, and immune dysregulation (**Figure 1**). With our advancing understanding of the complex interactions within these ecosystems, modulating the microbiome via interventions such as fecal microbiota transplantation (FMT), probiotics, prebiotics, and synbiotics holds growing promise for the treatment of ophthalmic diseases (85–88). Consequently, profiling and comparing the fecal and ocular microbiomic signatures of IBD patients with and without ocular involvement will be of significant clinical value.

**Figure 1:**
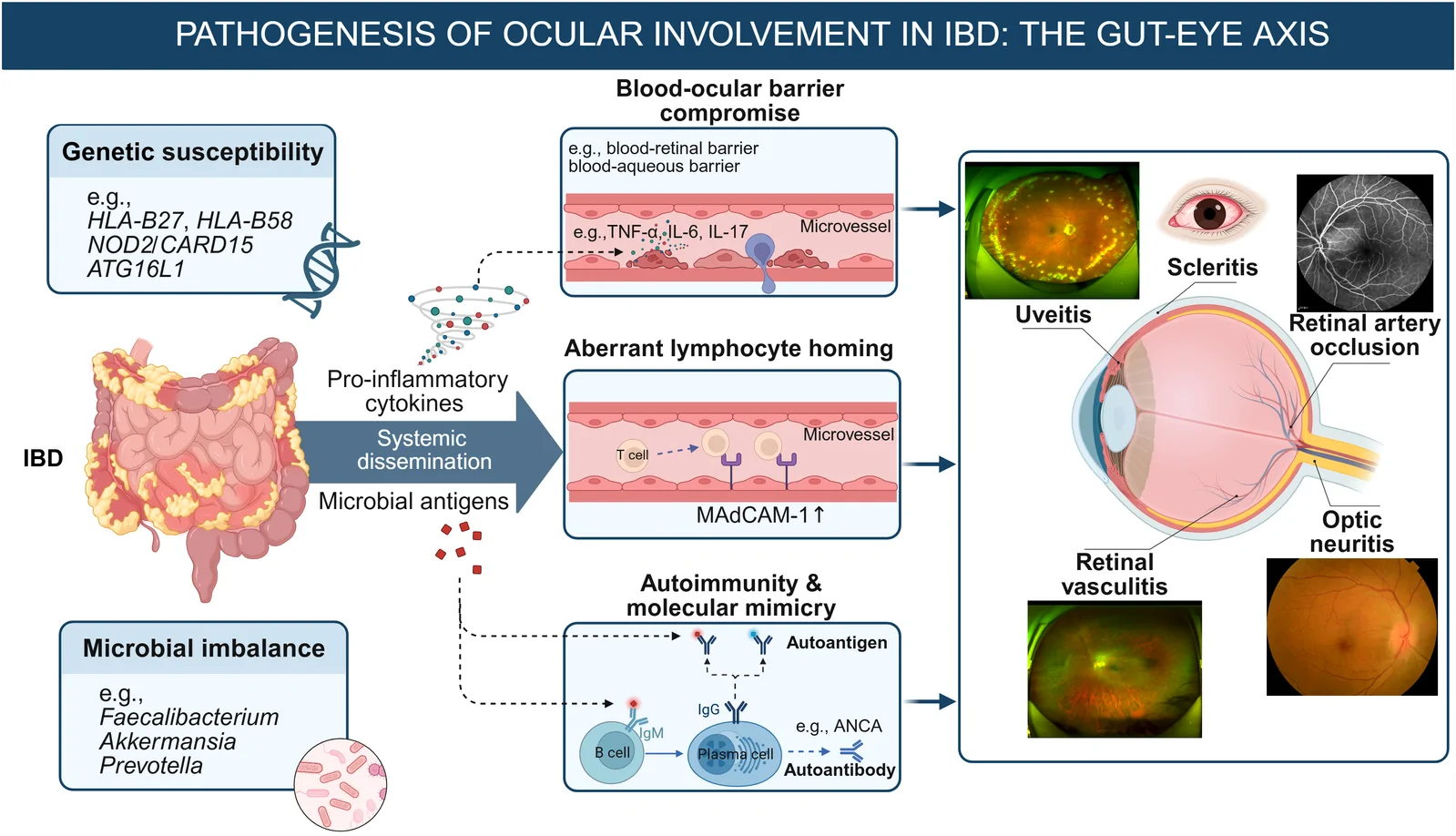
Pathogenesis of ocular involvement in IBD. The gut-eye axis in IBD is driven by a triad of immune dysregulation, genetic susceptibility, and microbiota dysbiosis.

## 5 Management of ocular complications in IBD

Management depends on whether the EIM parallels intestinal inflammation or follows an independent course, dictating whether treating the gut is sufficient or organ-specific therapy is required. The European Crohn’s and Colitis Organisation (ECCO) guidelines underscored the importance of a multidisciplinary approach when managing EIMs and suggested possible therapeutic strategies that could be used (89). Historically, managing IBD-associated ocular complications has been a clinical priority. Early literature showed that while corticosteroids alone benefited a minority of patients, the majority required tailored systemic therapies, such as NSAIDs and azathioprine, to preserve vision, especially in cases of CD or HLA-B27-positive anterior uveitis (90). Therapeutic strategies are largely dictated by the specific type of ocular involvement, its severity, and the activity of the underlying intestinal disease. Systemically directed anti-TNF-α therapies (infliximab, adalimumab, and certolizumab) possess the most robust evidence base for treating ocular EIMs. Meta-analyses indicate high response rates, with significant symptom improvement observed in the vast majority of patients suffering from uveitis, scleritis, and episcleritis. They remain the biologic treatment of choice for severe or refractory ocular manifestations (7, 91). Moreover, emerging non-invasive diagnostic tools are further refining this targeted approach. Recent studies demonstrate that optical coherence tomography can evaluate specific retinal and choroidal vascular parameters, such as the ganglion cell inner-plexiform layer and choroidal vascularity index. These optical coherence tomography-derived indices act as novel biomarkers that can highly predict not only IBD disease progression but also the clinical response to anti-TNF-α therapies, providing valuable visual guidance for biologic treatment (92). Prospective studies specifically involving patients with IBD-associated ocular inflammation are needed to validate their diagnostic and therapeutic utility.

Regarding anti-integrin therapy, vedolizumab presents a distinct clinical profile due to its gut-selective mechanism of action. Consequently, its ability to manage or prevent EIMs appears limited when compared to systemic treatments. A systematic review indicated a lack of strong evidence supporting vedolizumab for treating pre-existing EIMs (93). Furthermore, clinical data highlight a notable risk of EIM exacerbation during therapy. A meta-analysis of 21 studies reported that 28% of patients experienced EIM exacerbations (94). Similarly, data from the Spanish ENEIDA registry involving 551 patients revealed that vedolizumab exacerbated inactive EIMs in 13.5% of cases and induced *de novo* EIMs in 4.5%, which occasionally necessitated treatment discontinuation (95). When directly compared to anti-TNF therapies, the largest claims-based study found that patients with Crohn’s disease treated with vedolizumab were 28% more likely to develop new EIMs. Importantly, this included statistically significant increases in specific ocular complications such as episcleritis, scleritis, uveitis, and iritis (96). These findings suggest that while vedolizumab effectively controls intestinal inflammation, its gut-selective inflammatory control restricts its systemic efficacy, making it a suboptimal primary choice for patients with active or severe ocular manifestations. While robust evidence regarding the use of IL-23 and selective JAK inhibitors across EIMs is still limited, emerging data suggest differing clinical benefits. Systematic reviews of ocular EIMs indicate that ustekinumab, which targets the shared p40 subunit of interleukin (IL)-12 and IL-23, induces improvement in 55%–59% of patients with pre-existing uveitis, episcleritis, or scleritis, with a low incidence of *de novo* ocular disease (7). Selective IL-23 inhibitors, including risankizumab and mirikizumab, have demonstrated favorable long-term efficacy for dermatologic EIMs, particularly plaque psoriasis and other cutaneous manifestations; however, further studies are needed to establish their effectiveness in ocular EIMs (97). Real-world evidence suggests that JAK inhibitors are effective for several EIMs, particularly peripheral and axial spondyloarthritis and dermatologic manifestations; however, evidence specifically supporting their efficacy in ocular EIMs remains limited (98). Additionally, sphingosine 1-phosphate (S1P) receptor modulators require specific ophthalmologic consideration due to class-associated risks of macular edema. However, etrasimod, an oral selective S1P receptor modulator, has demonstrated a favorable safety profile; across multiple conditions, the incidence of macular edema and other ocular events in patients treated with etrasimod was uncommon and comparable to placebo (99). A comparative summary of the efficacy evidence, ocular safety considerations, and clinical implications of biologic and small-molecule therapies for ocular manifestations of IBD is provided in **Table 2**.

**Table 2:** Comparison of biologic and small-molecule therapies for ocular EIMs in IBD.

Therapeutic interventions	Drug class	Evidence for ocular EIMs
Infliximab, adalimumab, or certolizumab	Anti-TNF-α agents	Most robust evidence. High response rates have been reported for uveitis, scleritis, and episcleritis; considered the preferred biologic option for severe or refractory ocular EIMs.
Vedolizumab	Anti-integrin agent	Limited and inconsistent evidence. Its gut-selective mechanism may limit efficacy for active ocular EIMs; observational data suggest a risk of new-onset or exacerbated EIMs, including ocular manifestations.
Ustekinumab	IL-12/23 inhibitor	Moderate but limited evidence. Systematic reviews report improvement in 55%-59% of pre-existing uveitis, episcleritis, or scleritis, with few *de novo* ocular events.
Risankizumab and mirikizumab	Selective IL-23 inhibitors	Still limited. Evidence supports efficacy for dermatologic EIMs, but data specifically addressing ocular EIMs are insufficient.
Tofacitinib, upadacitinib, or filgotinib	JAK inhibitors	Still limited. Real-world studies suggest benefit for musculoskeletal and dermatologic EIMs; however, ocular EIM-specific efficacy data remain scarce.
Etrasimod	S1P receptor modulator	Still limited. Direct evidence for efficacy in ocular EIMs is lacking.

Evidence for ocular EIMs remains limited for most biologics and small-molecule therapies other than anti-TNF-α agents, partly owing to the relatively low prevalence and possible under-recognition of ocular EIMs in clinical practice.

The clinical management of specific ocular complications requires a highly coordinated effort and varies by condition. Because episcleral inflammation often mirrors intestinal disease activity, the primary therapeutic strategy for episcleritis is to adequately control the underlying IBD. Episcleritis is mostly self-limiting, and a conservative observational approach is generally advised, with topical corticosteroids being rarely necessary unless symptoms persist. Conversely, scleritis is a severe condition characterized by deep scleral inflammation, requiring immediate referral to an ophthalmologist to prevent permanent structural damage and vision loss. Its treatment typically involves topical anti-inflammatory agents, such as dexamethasone eye drops, while more severe cases necessitate systemic corticosteroids to achieve a favorable prognosis. Similarly, uveitis is a medical emergency that demands urgent ophthalmologic evaluation and intervention. While mild cases may be managed with topical corticosteroids, moderate to severe cases regularly necessitate step-up therapy utilizing systemic corticosteroids or systemic immunosuppressants. In these scenarios, anti-TNF agents are particularly valuable, as they effectively and simultaneously treat both intestinal and ocular inflammation (3, 100).

To facilitate timely referral and multidisciplinary care in real world settings, specific screening questionnaires can assist non-ophthalmologist physicians in identifying patients who require specialist evaluation. However, this represents a pragmatic recommendation, as no widely validated instrument is currently available specifically for routine screening of IBD-associated ocular inflammation. Creating an integrated clinical team is essential to improve IBD management, prevent disability, and improve patient quality of life (101). Ultimately, the current evidence guiding medical therapies for ocular complications in IBD remains heterogeneous and is generally of low quality, highlighting a significant need for prospective clinical trials to establish standardized, evidence-based treatment algorithms.

## 6 Conclusions and future directions

In conclusion, ocular manifestations are critical and potentially sight-threatening extraintestinal complications of IBD that underscore the systemic nature of the disease. The concept of the gut-eye axis provides a crucial framework for understanding how localized intestinal inflammation translates into distant ocular pathology through circulating inflammatory mediators, shared genetic susceptibilities, aberrant lymphocyte homing, and microbiota dysbiosis. The clinical management of these conditions dictates a highly individualized and multidisciplinary approach, closely involving both gastroenterologists and ophthalmologists. While anti-TNF agents remain the cornerstone for treating severe and refractory ocular EIMs, the advent of newer targeted therapies presents both opportunities and challenges. Specifically, the gut-selective mechanism of vedolizumab warrants cautious application in patients with active ocular inflammation, highlighting the necessity of tailoring biologic therapy to the patient’s comprehensive extraintestinal profile.

At present, studies concerning ocular manifestations in IBD still face several notable limitations. Current genetic insights into the causal links of IBD-associated ocular manifestations are largely derived from association studies or Mendelian randomization analyses, with direct mechanistic validation still lacking. However, the aforementioned basic research study provides a valuable research paradigm for investigating IBD-related ocular diseases (62). Additionally, there is a distinct lack of prospective, longitudinal studies explicitly linking intestinal inflammatory markers and mucosal healing directly to ocular outcomes. Epidemiological estimates are often hindered by selection bias, heterogeneous case definitions, and the absence of standardized, routine ophthalmologic screening in most large-scale IBD registries, which likely leads to the underreporting of subclinical ocular inflammation.

Research must bridge these critical knowledge gaps by shifting from purely associative observations to targeted translational investigations. Future research should prioritize the development of robust animal models for IBD-related ocular diseases, and comprehensively investigate the characteristics of the ocular and gut microbiomes, as well as ocular substructural parameters, in IBD patients with ophthalmic manifestations. Specifically, optical coherence tomography-derived retinal and choroidal indices could serve as dynamic biomarkers to predict disease progression and guide therapeutic decision-making, especially regarding responses to biologic therapies.

In the era of big data, the integration of artificial intelligence, particularly natural language processing, offers a transformative approach to characterizing EIMs in real-world cohorts (102, 103). By overcoming the limitations of structured electronic medical record data, automated natural language processing systems can efficiently identify ocular EIM profiles and clinical trajectories at the individual patient level. This technological advancement will facilitate larger scale, precise studies on shared immune mechanisms and personalized targeted therapies. Ultimately, well-designed prospective clinical trials are urgently needed to establish standardized, evidence-based treatment algorithms for ocular complications in IBD, ensuring optimal vision preservation and an improved overall quality of life for patients.
